# Ten-year survival after resection of a huge rhabdomyosarcoma in the pericardial cavity

**DOI:** 10.1186/s44215-024-00133-x

**Published:** 2024-03-04

**Authors:** Shohei Kitamoto, Yoichi Yamashita, Sayako Nakagawa, Taiko Horii

**Affiliations:** Department of Cardiovascular Surgery, Faculty of Medicine, Kagawa University, Kagawa, Japan

**Keywords:** Cardiac rhabdomyosarcoma, Long-term survival, Cardiac magnetic resonance

## Abstract

**Background:**

Primary malignant cardiac tumors are exceedingly rare and cardiac rhabdomyosarcoma among them is an exceptional rarity characterized by a dismal poor prognosis.

**Case presentation:**

A 48-year-old man had suffered from a persistent cough lasting for more than 6 months and computed tomography showed a huge mass in the pericardial cavity with heterogeneous content. Following referral to our department for suspected cardiac malignancy, cardiac magnetic resonance imaging revealed a lucent layer on the boundary around the mass, suggesting the feasibility of surgical resection. A baby-head-sized mass in the pericardial cavity was entirely resected and the pathological examination confirmed a tumor as a spindle cell type rhabdomyosarcoma. The patient underwent adjuvant chemotherapy and has well survived over a decade after surgery without any sign of recurrence.

**Conclusion:**

Radical resection with adjuvant chemotherapy may achieve a favorable outcome for patients with massive rhabdomyosarcomas inside the pericardial cavity.

## Background

Primary cardiac tumors are very rare diseases [1, 2] and one-quarter of primary cardiac tumors are malignant [3, 4]. Primary cardiac sarcoma is exceedingly rare with an extremely poor prognosis [5] and such scarcity makes cardiac surgeons hesitate to treat it properly.

## Case presentation

**Fig. 1:**
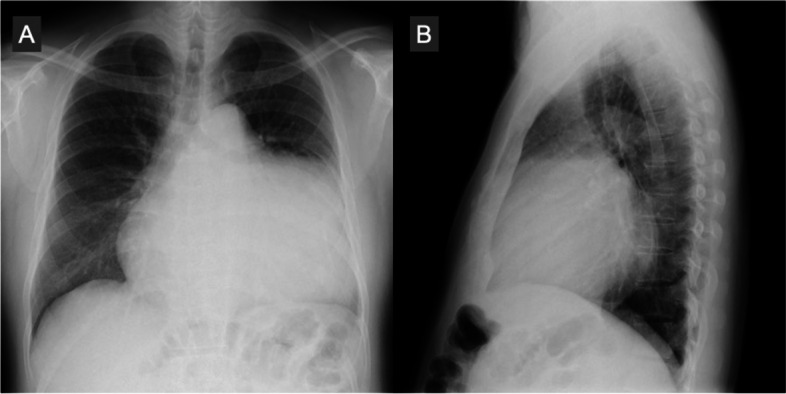
Preoperative chest X-ray. Chest X-ray shows cardiomegaly and a cardiac silhouette expands into the left thoracic cavity

**Fig. 2:**
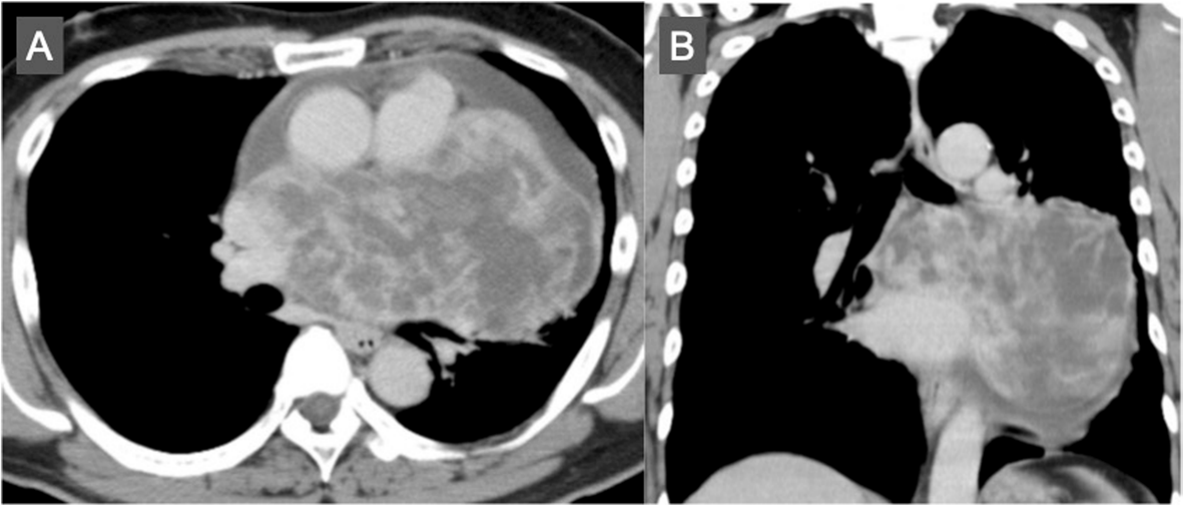
Preoperative computed tomography. A huge mass situates inside the pericardial cavity and compresses the heart. The content of the mass looks solid and heterogeneous with contrast medium

**Fig. 3:**
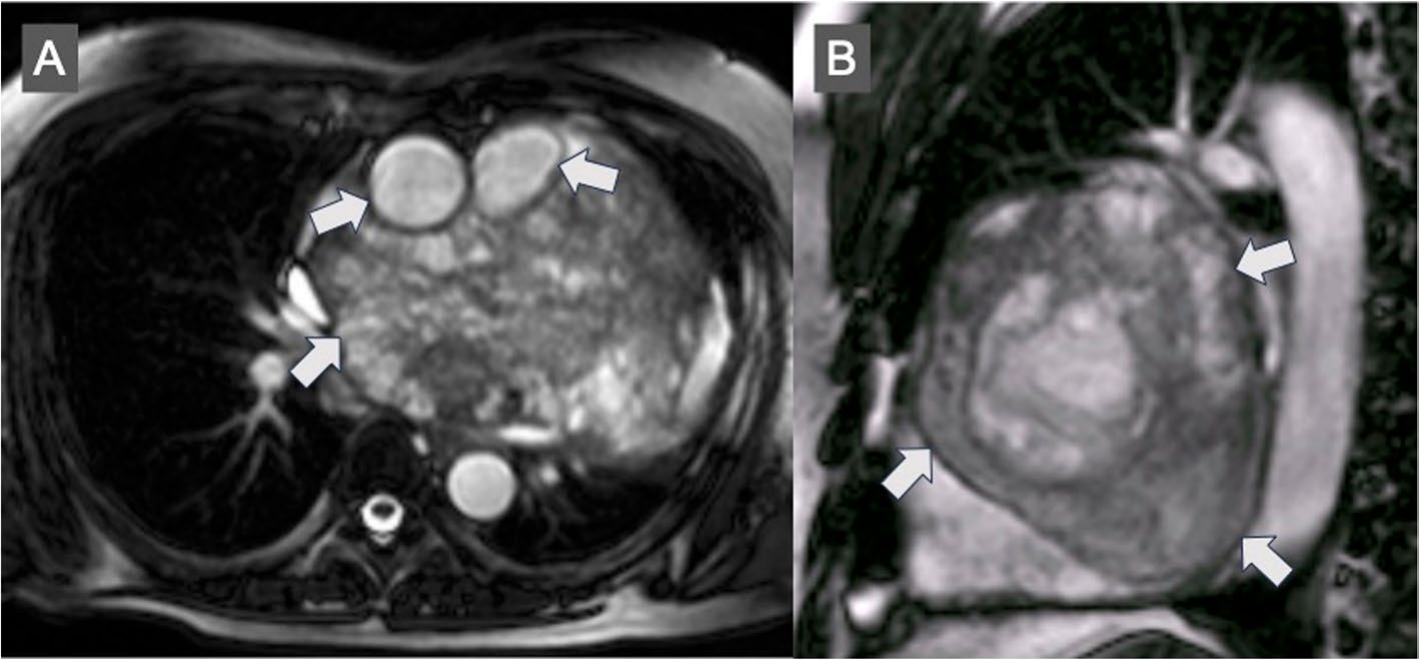
Preoperative cardiac magnetic resonance. Arrows reveal a lucent layer on the boundary around the huge mass

**Fig. 4:**
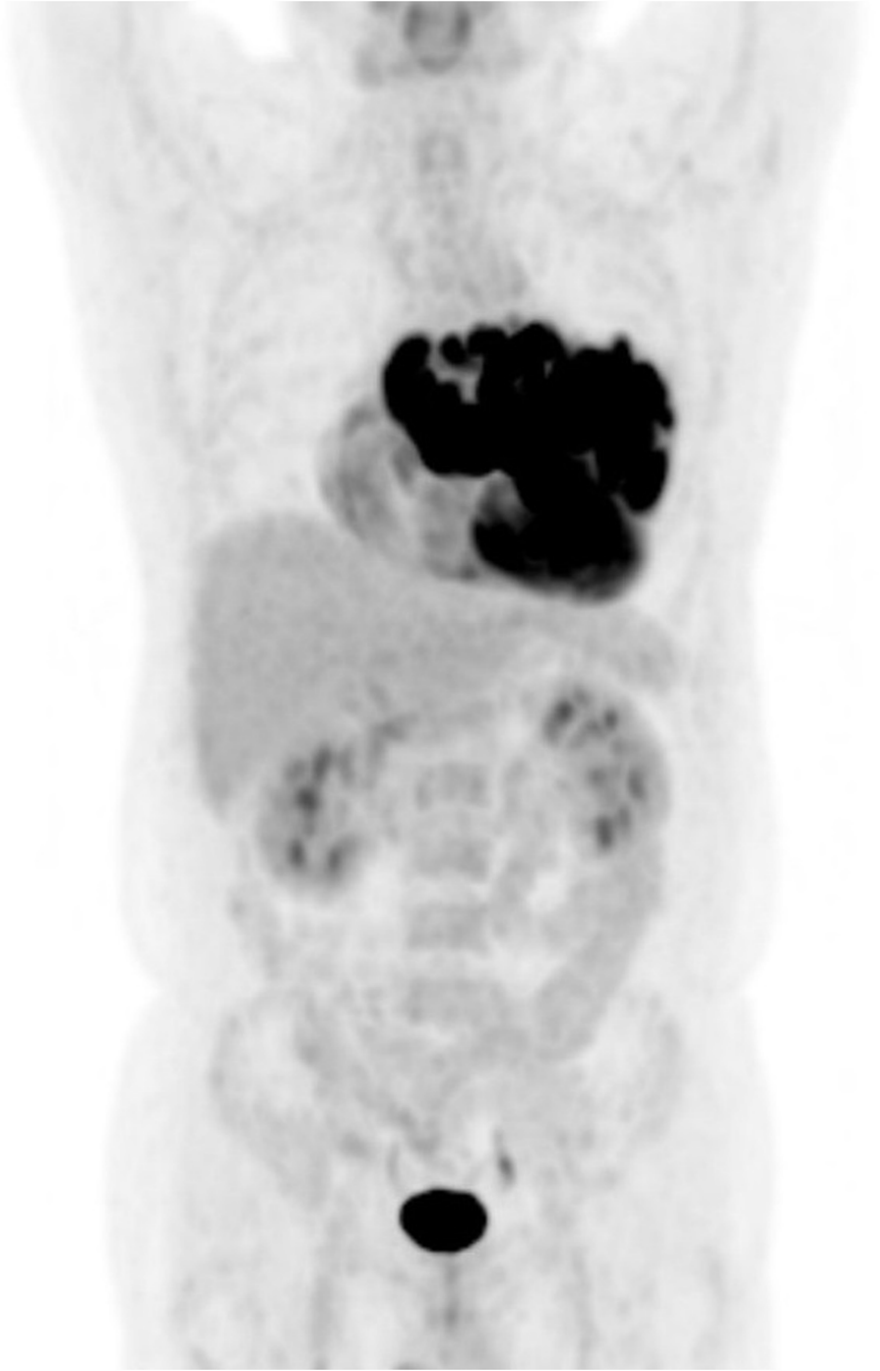
Preoperative positron emission tomography. Abnormal accumulations situate inside the pericardial cavity without any other findings

A 48-year-old man suffered from a persistent cough lasting for more than 6 months and consulted a medical service. Cardiomegaly on chest roentgenogram (Fig. 1a, b) brought the patient to the general hospital for further assessment and computed tomography (CT) imaging revealed a huge mass situated inside the pericardial cavity and compressed the heart (Fig. 2a, b). The patient was subsequently referred to our department for comprehensive evaluation and treatment. CT imaging showed a baby-head-sized mass located behind and to the left of the heart and the content of the mass was solid and heterogenous with contrast medium. These radiological findings raised concerns regarding malignancy. An echocardiogram presented that a huge mass heavily compressed the heart and the left ventricle (LV) ejection fraction was reduced down to 30% with akinesis of the antero-septal area. However, no definitive evidence of LV wall invasion or intracavitary protrusion of the mass was observed on echocardiography and CT. In order to examine the extension of this tumor we obtained cardiac magnetic resonance imaging (CMR). CMR revealed a lucent layer on the boundary around the mass, hinting at the potential for surgical dissection from surrounding cardiovascular structures such as the heart and the great vessels (Fig. 3a, b). ^18^F-fluorodeoxyglucose positron emission tomography coupled with CT (FDG PET-CT) failed to detect any additional abnormal findings beyond the intrapericardial mass (Fig. 4).

**Fig. 5:**
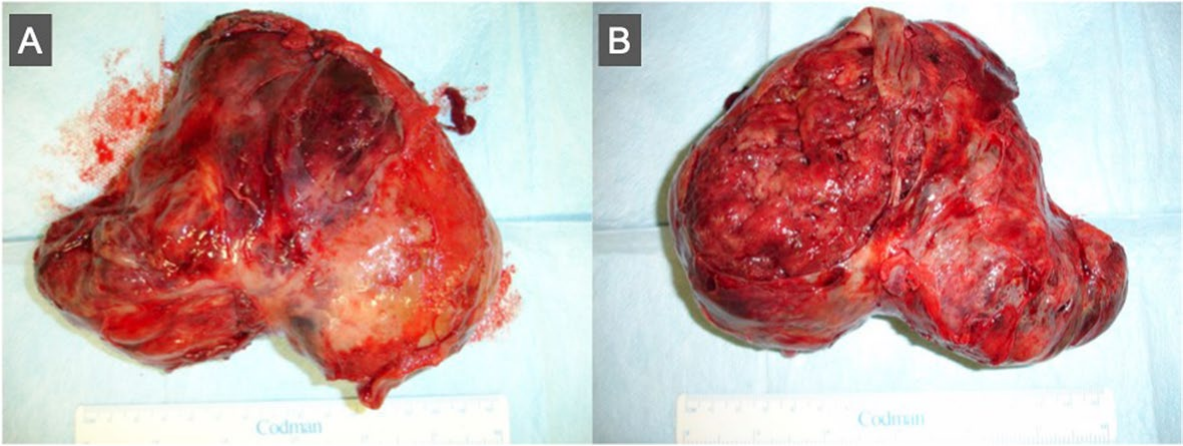
Gross appearance of the tumor. A baby head-sized tumor was 20 × 25 × 15 cm with 600 g in weight

Initially, oncologists proposed a percutaneous biopsy to facilitate the diagnostic and therapeutic process, with chemotherapy or radiotherapy; however, the physical status of the patient was relatively good enough to tolerate more invasive treatment. We made a decision to proceed with a median sternotomy for excising a specimen from a tumor or for resecting a whole mass if possible. After opening the pericardium, the surface was relatively smooth and monotonous without any sign of a mass behind the heart. Encouraged by the presence of a lucent layer as shown in CMR, we initiated a careful dissection in the space between the aorta and the superior vena cava with HARMONIC SYNERGY ultrasonic blade (Ethicon, CO, USA) and the dissection extended posteriorly toward the rear of the heart. The adhesion between the surface of the mass and the surrounding tissues displayed characteristics with loose connective tissue, reminiscent of the adhesion frequently encountered during redo surgeries. The heart with reduced LV ejection fraction was so well tolerated to be lifted with apical sucking by a vacuum system that we could separate the cardiovascular component including the heart and the great vessels free from a mass without utilizing the cardiopulmonary bypass circuit. The ventral part of a huge mass was able to be resected smoothly without any damage to its capsule, but the dorsal part was quite difficult to resect bluntly. A small segment densely adhered to the ventral surface of the left atrium near the left pulmonary vein and was unable to be free. We utilized a cardiopulmonary bypass and resected a huge mass en bloc in a forced fashion on-pump beating heart, then a hole around 2 cm in diameter close to the conjunction of the left atrium and the left superior pulmonary vein was repaired with autologous pericardium. The cardiopulmonary bypass could be weaned off in 60 min uneventfully. A kidney-shaped mass entirely capsulated was 20 × 25 × 15 cm in size and 600 g in weight (Fig. 5a, b). The postoperative course was not straightforward with temporary convulsion and sedation for 72 h, then the patient fully recovered without any neurological disorder and was discharged on postoperative day 30.

Pathological examination confirmed spindle cell type rhabdomyosarcoma by immunohistopathological test with staining desmin, myogenin, and MYOD 1. Gross examination revealed a well-capsulated tumor with a focal disruption of the capsule at the posterior surface, indicating contact with the left atrial wall. The tumor presumably originated from the epicardial wall of the left atrium and expanded outward to the pericardial cavity. The patient underwent postoperative chemotherapy, receiving a full dose of cisplatin and pirarubicin as recommended by our institutional oncologist. Subsequent echocardiography demonstrated a remarkable recovery in LV ejection fraction from 30% before surgery to 60% at 6 months after surgery enabling the patient to return to full occupational activity.

**Fig. 6:**
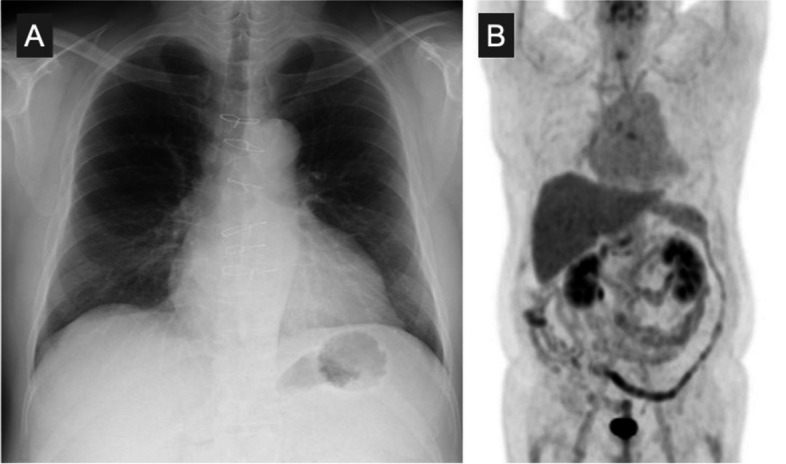
Postoperative image at 10 years after resection. **A** Chest X-ray shows that the cardiac silhouette shrinks down into the normal range. **B** Positron emission tomography reveals no abnormal accumulation

Ten years following the successful surgical removal of cardiac rhabdomyosarcoma, the patient has maintained an active and unrestricted social life. A 10-year follow-up FDG PET-CT confirmed the absence of any signs of recurrence (Fig. 6).

## Discussion and conclusion

Primary cardiac tumors are very rare diseases [1], with an autopsy incidence of 0.02% [2], and one-quarter of primary cardiac tumors are malignant [3, 4]. Primary cardiac sarcoma is exceedingly rare with a poor prognosis and has a median survival of 7 months [5]. Most cardiac surgeons do not have enough knowledge and experience to treat cardiac malignancies properly. A few surgeons have encountered some cases by chance and the outcome varied widely from relatively mild consequence to rapid deterioration immediately even after radical resection [5].

Rhabdomyosarcoma (RMS) is the most common soft tissue tumor of the pediatric age and can be cured in nonmetastatic disease by utilizing multimodality treatment, combining complete resection, chemotherapy, and radiotherapy [6]. Contrary to the pediatric age, RMS in adults is exceedingly rare with a very poor prognosis and such a multidisciplinary approach as in the pediatric age is recommended to improve the outcome of RMS in adults [7]. Surgery and chemotherapy have a positive impact on the survival of primary cardiac sarcoma but increasing age is associated with worse survival [5]. The patient with a huge RMS inside the pericardial cavity of middle age must be moribund with rapid deterioration even if a multimodal approach could be fully applied. In our case, there were not only bad factors such as size, age, and location, but also good factors as nonmetastatic as confirmed by FDG PET-CT. The reason why our cardiac rhabdomyosarcoma did not invade surrounding tissues remains unclear. Complete resection is pivotal in achieving a good prognosis and even incomplete resection with maximizing volume reduction of a huge mass may contribute to a better outcome than just a biopsy with a whole mass left behind [5].

CT imaging initially provided limited insight, rendering obscure images on the boundary between the mass and adjacent cardiovascular structures. The diagnostic potential of CT was constrained, and no definitive perspective was attainable. Subsequent CMR imaging presented a lucent layer around the mass, inspiring confidence in the feasibility of mass resection and steering us toward a more aggressive surgical approach. When we opened the pericardium, the outlook was so monotonous but CMR images helped us to proceed further to remove a tumor. CMR played a pivotal role in our case by aiding in both diagnosis and surgical planning, particularly in the context of primary cardiac masses.

In conclusion, a huge rhabdomyosarcoma located within the pericardial cavity was successfully resected and the combined approach of radical resection and adjuvant chemotherapy has enabled the patient to achieve over a decade of recurrence-free survival. The presence of the lucent layer on the boundary around the mass unveiled by cardiac magnetic resonance encouraged us to resect a whole mass rather than excise a specimen just for the diagnosis.

## Data Availability

Data of this report is available from the corresponding author upon reasonable request. No datasets were generated or analyzed during the current study.

## Abbreviations

CT: Computed tomography
CMR: Cardiac magnetic resonance
FDG PET-CT: Fluorodeoxyglucose positron emission tomography
LV: Left ventricle

## References

[1] Amano A, Nakayama J, Yoshimura Y, IkedaU. Clinical classification of cardiovascular tumors and tumor-like lesions, and its incidences. Gen Thorac Cardiovasc Surg. 2013;61:435–47. 23460447 10.1007/s11748-013-0214-8 PMC3732772

[2] Reynen K. Frequency of primary tumors of the heart. Am J Cardiol. 1996;77:107. 8540447 10.1016/s0002-9149(97)89149-7

[3] Burke A. Primary malignant cardiac tumors. Semin Diagn Pathol. 2008;25:39–46. 18350921 10.1053/j.semdp.2007.10.006

[4] Campisi A, Ciarrocchi AP, Asadi N, Dell’Amore A. Primary and secondary cardiac tumors: clinical presentation, diagnosis, surgical treatment, and results. Gen Thorac Cardiovasc Surg. 2022;70:107–15. 35000140 10.1007/s11748-021-01754-7

[5] Yin K, Luo R, Wei Y, Wang F, Zhang Y, Karlson KJ, et al. Survival outcomes in patients with primary cardiac sarcoma in the United States. J Thorac Cardiovasc Surg. 2021;162:107–15. 32111430 10.1016/j.jtcvs.2019.12.109

[6] Crist WM, Anderson JR, Meza JL, Fryer C, Raney RB, Ruymann FB, et al. Intergroup rhabdomyosarcoma study-IV: results for patients with nonmetastatic disease. J Clin Oncol. 2001;19:3091–102. 11408506 10.1200/JCO.2001.19.12.3091

[7] Fischer TD, Gaitonde SG, Bandera BC, Raval MV, Vasudevan SA, Gow KW, et al. Pediatric-protocol of multimodal therapy is associated with improved survival in AYAs and adults with rhabdomyosarcoma. Surgery. 2018;163:324–9. 29217286 10.1016/j.surg.2017.10.027

